# Epigenetic regulation of ID4 in breast cancer: tumor suppressor or oncogene?

**DOI:** 10.1186/s13148-018-0542-8

**Published:** 2018-08-23

**Authors:** Daniela Nasif, Emanuel Campoy, Sergio Laurito, Richard Branham, Guillermo Urrutia, María Roqué, María T. Branham

**Affiliations:** 1 IHEM, National University of Cuyo, CONICET, Mendoza, Argentina; 2 IHEM, Faculty of Exact and Natural Sciences, National University of Cuyo, CONICET, Mendoza, Argentina; 3 IHEM, CONICET, Facultad de Ciencias Médicas, National University of Cuyo, Mendoza, Argentina; 4 IANIGLA, CONICET, Mendoza, Argentina

**Keywords:** ID4, Tumor suppressor, Breast cancer, Methylation

## Abstract

**Background:**

Inhibitor of differentiation protein 4 (ID4) is a dominant negative regulator of the basic helix-loop-helix (bHLH) family of transcription factors. During tumorigenesis, ID4 may act as a tumor suppressor or as an oncogene in different tumor types. However, the role of ID4 in breast cancer is not clear where both an oncogenic and a tumor suppressor function have been attributed. Here, we hypothesize that ID4 behaves as both, but its role in breast differs according to the estrogen receptor (ER) status of the tumor.

**Methods:**

ID4 expression was retrieved from TCGA database using UCSC Xena. Association between overall survival (OS) and ID4 was assessed using Kaplan–Meier plotter. Correlation between methylation and expression was analyzed using the MEXPRESS tool. In vitro experiments involved ectopic expression of ID4 in MCF-7, T47D, and MDA-MB231 breast cancer cell lines. Migration and colony formation capacity were assessed after transfection treatments. Gene expression was analyzed by ddPCR and methylation by MSP, MS-MLPA, or ddMSP.

**Results:**

Data mining analysis revealed that ID4 expression is significantly lower in ER+ tumors with respect to ER− tumors or normal tissue. We also demonstrate that ID4 is significantly methylated in ER+ tumors. Kaplan–Meier analysis indicated that low ID4 expression levels were associated with poor overall survival in patients with ER+ tumors. In silico expression analysis indicated that ID4 was associated with the expression of key genes of the ER pathway only in ER+ tumors. In vitro experiments revealed that ID4 overexpression in ER+ cell lines resulted in decreased migration capacity and reduced number of colonies. ID4 overexpression induced a reduction in ER levels in ER+ cell lines, while estrogen deprivation with fulvestrant did not induce changes neither in ID4 methylation nor in ID4 expression.

**Conclusions:**

We propose that ID4 is frequently silenced by promoter methylation in ER+ breast cancers and functions as a tumor suppressor gene in these tumors, probably due to its interaction with key genes of the ER pathway. Our present study contributes to the knowledge of the role of ID4 in breast cancer.

**Electronic supplementary material:**

The online version of this article (10.1186/s13148-018-0542-8) contains supplementary material, which is available to authorized users.

## Background

Inhibitor of differentiation (ID) proteins 1, 2, 3, and 4 regulate the expression of genes by acting as dominant negative regulators of the basic helix-loop-helix (bHLH) transcription factors. ID proteins interact with the bHLH transcription factors and form heterodimers, inhibiting in this way their possible binding to DNA since ID proteins lack the basic DNA-binding domain [1]. During differentiation, the expression of ID proteins is downregulated in cells, and on the contrary, it is increased in stem cells [2]. In human tumors, an increased expression of ID proteins has been associated with reversion to an embryonic-like state, with loss of differentiation, increased migration, proliferation, and neo-angiogenesis [3]. However, discordant literature attributes opposite roles to ID proteins; for example, some studies have also recognized ID proteins as critical actors of antiproliferative signaling pathways in cancer [4]. So, it seems then that, according to the cellular context, ID proteins can pursue divergent functions and act as oncoproteins or tumor suppressors [2].

ID4 is a member of this protein family and it has been shown to be highly expressed in neurons [5], osteoblasts [6], adipocytes [7], prostate epithelial cells [8], and testicular Sertoli cells [9]. In embryogenesis, ID4 is required for normal mammary [10] and prostate gland development [8]. In cancer, ID4 presents again divergent roles since it has been described to act as a tumor suppressor in prostate [11], lung [12], and gastric [13] tumors, and as an oncogene in ovarian cancer [14] and glioblastomas [15].

In breast cancer, the role of ID4 is not clear. Epigenetic silencing of ID4 (a characteristic mechanism to downregulate tumor suppressor genes during cancer progression) has been described in mammary columnar cell lesions, ductal carcinoma in situ, and invasive carcinomas [16]. In addition, the hypermethylation of ID4 promoter has been associated with an increased risk of lymph node metastasis [17]. Epigenetic silencing and gene expression downregulation are hallmarks of tumor suppressor gene function, since their absence allows the progress of a tumorigenic process. However, we and others have found the opposite role for ID4 in breast tumors. Increased ID4 expression has been informed in basal cell-like breast cancer [18], and we found increased expression and hypomethylation of its promoter in triple-negative breast cancer (TNBC) [19]. Moreover, increased ID4 expression has been associated with the ability of breast cancer cells to exhibit anchorage-independent growth [20]. Also, high ID4 expression in TNBC has been associated with BRCA1 down regulation and BRCAness phenotype [21, 22]. Therefore, enough evidence exists to conclude that ID4 can assume distinct roles in breast cancer, depending on the cellular context. We hypothesize that ID4 acts as both, tumor suppressor and oncogene, but its role will differ according to the ER status of the breast cancer cell. We have previously demonstrated that ID4 acts as an oncogene in ER-negative tumors. In this work, we hypothesize that ID4 may behave as a tumor suppressor in an ER+ cellular context.

## Methods

### Cell lines and cell culture

Human breast cancer cell lines MCF-7, T47D, and MDA-MB231, were kindly provided by Dr. Lanari from the IBYME Institute, Buenos Aires, Argentina and by Dr. Matias Sanchez, IMBECU Institute, Mendoza, Argentina, respectively. Cell lines were cultured in DMEM medium (Gibco by Life Technologies, Grand Island, NY, USA, # 1852779) supplemented with 10% fetal bovine serum (Internegocios S.A, Mercedes, BA, Argentina), 100 U/mL of penicillin, and 100 μg/mL streptomycin (Gibco by Life Technologies, Grand Island, NY, USA, #1796440). All cell lines were incubated at 37 °C in a humidified atmosphere containing 5% CO_2_. For estrogen depletion experiments, cells were cultured in phenol red-free RPMI supplemented with charcoal-stripped 10% fetal bovine serum for 1 week prior to drug treatment. After this time, fulvestrant 1 μM was added to the medium and cells were treated for 72 h.

### Plasmids and transfections

The full-length human ID4 cloned into pCMV vector (pCMV-Id4) was a generous gift from Dr. Mark Israel. Transfection of 3 μg of pCMV-ID4 or 3 μg pGFP (as control vector) were performed with Lipofectamine 2000 (Invitrogen, Van Allen Way Carlsbad, CA, USA # 1828126) at 90% confluence according to the manufacturer’s instructions. Transfection was monitored by fluorescence microscopy and after 48 h, it achieved a 70% efficiency. ID4 overexpression was confirmed by Western blot (Additional file 1: Figure S1). After transfection, different assays were performed as described below.

### Migration assay

Forty-eight hours after transfection, the migration assay was started. Cells were serum-starved overnight before the scratch was produced; afterwards, the cells were maintained in a serum-reduced medium containing 0.5% FBS. Cell cultures were then scratched with a 200 μL sterile pipette tip and extensively washed with PBS to remove detached cells and debris. One cross was scratched in each well; then, images of the same area were taken at 24, 48, and 72 h. These were instantly center-imaged at × 4 magnification, using a T-2000 microscope equipment (Nikon, Tokyo, Japan).

### Colony formation assay

After transfection, MCF7 and T47D cells were platted at a density of 1000 cells per well in six well plates and allowed to adhere overnight at 37 °C, 5% CO_2_. The cells were allowed to grow until control treatment colonies reached > 50 cells per colony (approximately 12 days). Colonies were then fixed with glutaraldehyde for 30 min, stained with crystal violet 0.5% for 30 min and washed. Next, colony number was counted by using an automatized procedure with Image J software.

### Methylation analysis

For MSP and droplet digital MSP (ddMSP) assays, DNA was firstly bisulfite-converted with the EZ DNA Methylation-Direct ™ Kit (Zymo RESEARCH, Irvine, California, USA). Primers used for MSP and ddMSP were specific for detection of the methylated and un-methylated status of the ID4 promoter. Primers were purchased from Integrated DNA Technologies (CA, USA). Forward primers for MSP covered the TATA box, E-box, and three CpG sites in the minimal promoter region (− 48 to + 32) [23]. The methylation-specific primer set was as follows: forward, 5′-TTTTATAAATATAGTTGCGCGGC-3′; and reverse, 5′-GAAACTCCG ACTAAACCCGAT-3′. The unmethylation-specific primer set was as follows: forward, 5′-TTT TATAAATATAGTTGTGTGGTGG-3′; and reverse, 5′-TCA AAACTCCAACTAAACCCAAT-3′. The PCR amplification reaction for MSP was performed in a 25 μl final volume and consisted of a 40-cycle program, composed of 30s at 94 °C, 30 s at 58 °C, and 30 s at 72 °C, followed by a 7 min final extension at 72 °C. Mg^2+^ concentration was 1.5 mM for methylated-specific and 2.5 mM for unmethylated-specific primer sets. Primer concentration was 0.1 μM for methylated-specific and 0.4 μM for unmethylated-specific primer sets. PCR products were resolved by 2% agarose gel electrophoresis and quantified by ImageJ software. ddPCR general specifications are described below.

The MS-MLPA assays were performed basically according to manufacturer’s recommendations (MRC-Holland, Amsterdam, The Netherlands, (www.mlpa.com). A CpG site was considered to be methylated when the methylation dosage ratio between digested and undigested sample was superior to the cut-off threshold of 8% [19].

### Droplet digital PCR

Purified RNA was converted to cDNA using M-MLV Reverse Transcriptase, and cDNA was diluted to 0.2 ng/μl and stored at − 20 °C until use. For each assay, 5 μl of the diluted sample (1 ng cDNA) was run using the Bio-Rad EvaGreen master mix. For each assay, droplets were generated using Droplet Generation Oil for EvaGreen (Bio-Rad) on the QX200 Droplet Generator (Bio-Rad) according to the manufacture’s protocol and adding the specific primers for ER with the following sequence: forward, 5′-CAGGACTCGGTGGATATGGT; and reverse, 5′-CCAGGGAAGCTACTGTTTGC. Droplets were cycled on the C1000 Touch Thermal Cycler (Bio-Rad) for 40 cycles, with a 58 °C annealing temperature. Droplets were read using the QX200 Droplet Reader (Bio-Rad). Data was analyzed in QuantaSoft software (Bio-Rad). Each DNA sample was run in three technical replicates and the mean was considered for further comparisons.

### In silico data analysis

TCGA breast cancer data was obtained from the UCSC Xena resource (http://xena.ucsc.edu/). For gene expression, the RNA-Seq (polyA+ Illumina HiSeq) data was downloaded as log2 (norm_count+ 1) values. For methylation analysis, the Illumina Infinium Human Methylation 450 platform was retrieved. This platform represents DNA methylation as beta values, which are continuous variables between 0 and 1, representing the ratio of the intensity of the methylated bead type to the combined locus intensity.

Additionally, the MEXPRESS tool was used for visualization and interpretation of the expression, methylation, and clinical data available in TCGA (http://mexpress.be/) [24]. Survival curves were estimated using the Kaplan–Meier plotter tool (http://kmplot.com/analysis/). This tool uses an online database of published microarray datasets for breast, ovarian, lung, and gastric cancer, and it includes clinical and gene expression data for 5143 breast cancer patients [25]. The analyses were performed using the JetSet best probe set. Survival was also analyzed using the breast cancer Miller cohort downloaded form (http://xena.ucsc.edu/) [26].

The association between relative gene expression values was performed by SVD (singular-value decomposition) as previously described [22]. The correlation coefficients between ID4 and each specific gene were calculated by the standard statistical procedure described by Wonnacott and Wonnacot (Introductory Statistics, 2nd ed., John Wiley, 1972, pp. 326–331). These statistical analyses were performed using MATLAB (Natick, MA, USA). mRNA expression differences between groups of breast cancers were assessed using unpaired Student’s *t* test and a one-way analysis of variance with Bonferroni’s post hoc analysis for comparison between multiple groups.

#### ArrayExpress data

For ID4 expression analysis in MCF-7 control vs. fulvestrant-treated cells, an in vitro model of MCF-7 cells treated with ER antagonists was retrieved from ArrayExpress with the accession number E-MTAB-4426. The intensity of ID4 probe (A_23_P59375) was compared between the following conditions: WT_CCS+ Fulv vs. WT_CCS + DMSO [27].

### Additional statistical analysis

Unless otherwise noted, all laboratory experiments were realized a minimum of three separate times and statistical analysis were performed using Graph Pad Prism software version 5. A Student’s *t* test was used for comparison between two groups. Significance was defined as a *p* value < 0.05.

## Results

### In silico analyses reveal that ID4 expression differs according to ER status in breast cancer

**Fig. 1:**
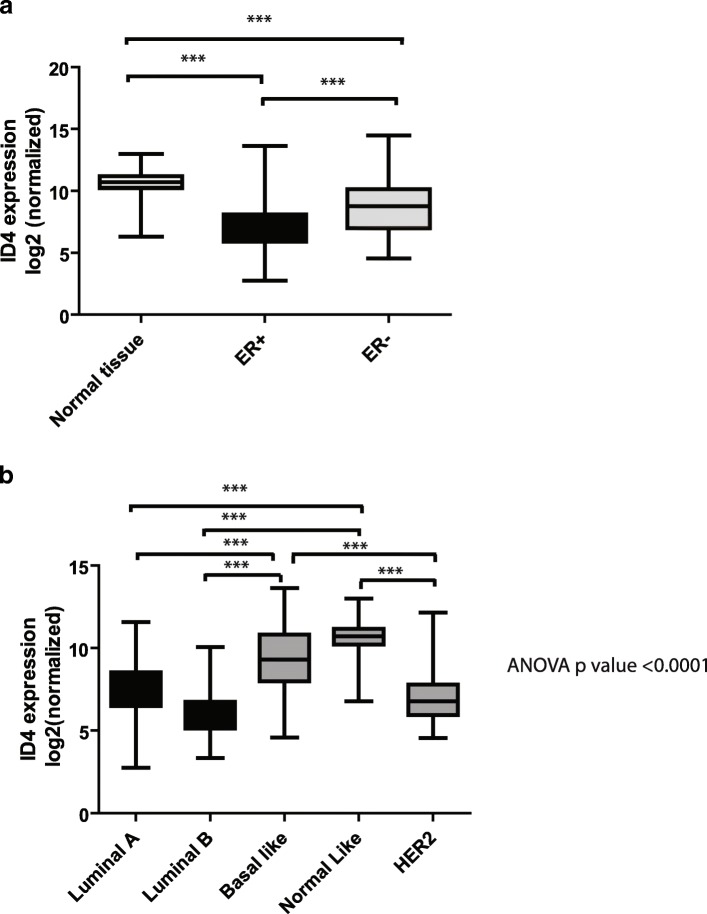
Comparison of ID4 expression among different breast cancer subtypes. **a**–**b** The expression of ID4 is shown relative to **a** ER+ (*n* = 601) and ER− (*n* = 179) and normal tissue (*n* = 114), **b** PAM50 molecular subtypes: luminal **a** (*n* = 434), luminal **b** (*n* = 194), basal-like (*n* = 142), normal-like breast tumors (*n* = 119), and HER2-enriched (*n* = 66). The Student’s *t* test was applied to evaluate differences in ID4 expression between two groups and one way analysis of variance (ANOVA) with Bonferroni’s post hoc analysis to compare three or more groups. The bottom and top of the box represent the first and third quartiles of the data, respectively, and the band inside the box represents the median of the data. The lower and upper whiskers represent the lowest and highest data points of the data, respectively. As can be seen in panel **a** and **b**, ID4 expression is reduced in ER+ subgroups ***p < 0.001

We first aimed to study the expression of ID4 across breast tumors with different ER status. For this, we used the IlluminaHiSeq_RNASeqV2 expression data from 780 breast tumors and 138 normal breast tissue samples from the TCGA (The Cancer Genome Atlas) database. We divided the tumor samples in two groups: ER+ (*n* = 601) and ER− (*n* = 179). As shown in Fig. 1a, ER+ breast tumors present a significant reduction in ID4 expression as compared to ER− tumors and to normal tissue (*p* < 0.001). Next, we performed a new analysis but, in this case, considering ID4 expression according to the PAM50 breast cancer molecular classification, i.e., luminal A, luminal B, normal-like, basal-like, and HER2-enriched. As shown in Fig. 1b, ID4 expression is significantly lower in luminal A (*n* = 434) and luminal B (*n* = 194) subtypes (both ER+) as compared with basal-like (*n* = 142) and normal-like breast tumors (*n* = 119) (both ER−) (*p* < 0.001). HER2-enriched subtype (*n* = 66) showed significantly lower levels of ID4 expression with respect to basal-like or normal-like tumors (*p* < 0.001). This observation could be confusing given that this subtype is often thought of as being ER− only. However, it should be taken into consideration that the HER2-enriched subtype can include ER+ and ER− tumors as well. This mixed composition could explain lower levels of ID4 expression for this subtype.

Taken together, our results show that ID4 expression differs according to the ER status, and that its expression is significantly lower in ER+ breast tumors.

### High ID4 expression is associated with better prognosis in ER+ breast tumors

**Fig. 2:**
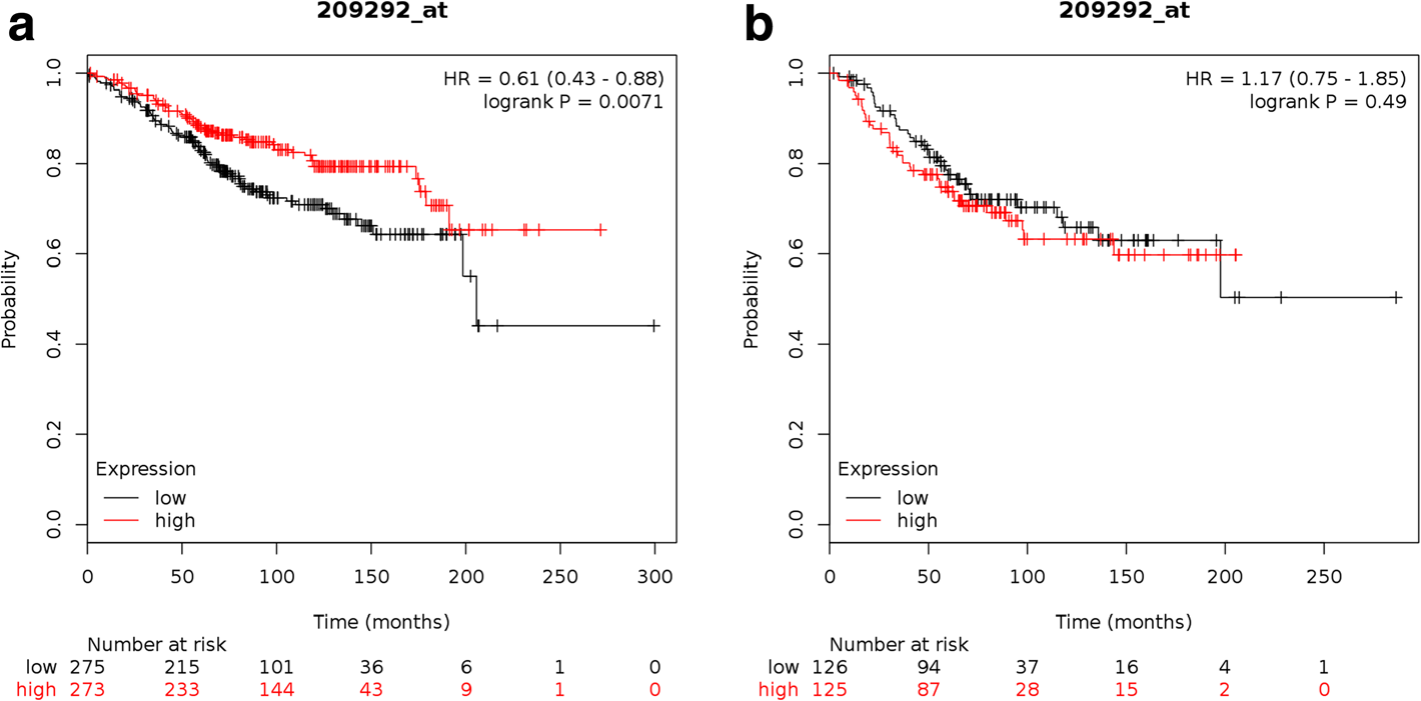
Kaplan–Meier survival curves indicate a better survival for high ID4 expression in patients with ER+ tumors. **a**–**b** Overall survival curves calculated by KM plotter, for patients with tumors classified as ER+ (**a**) and ER− (**b**) respectively. Survival probability is represented on the y-axis, time (in months) on the x-axis. Black curve corresponds to low ID4 expression, and red curves to high ID4 expression. As can be noticed, only in a ER+ context the enhanced expression of ID4 contributes to a difference in OS (panel **a**)

To evaluate whether the expression level of ID4 could have any predictive value for breast cancer overall survival (OS), we used the online survival analysis software, Kaplan–Meier (KM) plotter [25]. This tool allowed us to study the expression of ID4 as dichotomized values in “high” or “low” according to the median expression of the gene. The relationship between ID4 expression and OS of 799 breast cancer patients was analyzed separating ER+ (*n* = 548) from ER− (*n* = 251) cases. As shown in Fig. 2a, among the patients with ER+ tumors, those with higher ID4 expression levels presented better probabilities of survival (*p* < 0.001); this result suggests that this group of patients with high ID4 expression levels have an active (not silenced) ID4 tumor suppressor gene. Remarkably this was not observed in ER− tumors (Fig. 2b) (*p* = 0,49). To confirm these results, we performed a Kaplan–Meier analysis using another database such as the Miller 2005 cohort. We analyzed OS in 213 ER+ breast cancer patients dividing ID4 values in high or low according to the median expression of the gene. As shown in Additional file 2: Figure S2 higher ID4 expression levels were associated with better probabilities of survival in line with Kaplan–Meier plotter results. Given that high ID4 expression was only beneficial in the ER+ group, we speculate that ID4 has a tumor suppressor role in these tumors.

### ID4 expression is downregulated through methylation in ER+ breast tumors as assed by in silico analyses

**Fig. 3:**
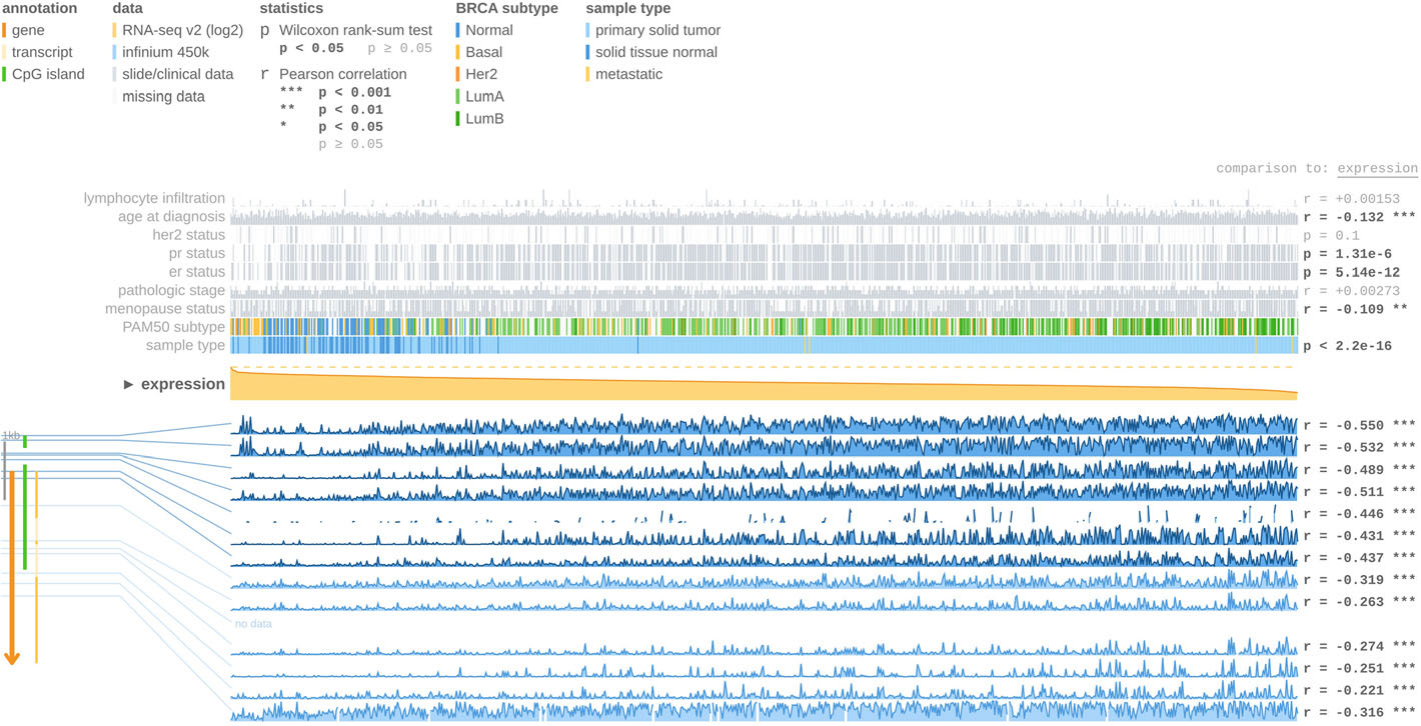
ID4 expression and methylation status in breast cancer using MEXPRESS. At the top of the figure clinical TGCA data available is represented and ordered according to ID4 expression. At the right-hand side, the Pearson’s correlation coefficient *r* and the *p* values for Wilcoxon rank-sum test are shown. ID4 expression is symbolized as the orange line in the center of the plot. The samples are ordered according to ID4 expression, with the highest expression on the left side and the lowest on the right. The blue lines (bottom right) represent the Infinium 450 k probes that are linked to ID4. The height of the blue lines indicates the beta value for the probe. The probes localized in the promoter region of the gene are highlighted in dark blue. ID4 gene and CpG islands (green lines) are represented on the left (bottom)

Since ID4 expression has been shown to be principally regulated trough methylation [13, 17, 28], we asked if there are differences in the methylation levels of ID4 according to the ER status. To answer this question, we queried the MEXPRESS tool which allows the visualization and interpretation of the gene expression, the methylation and the clinical data available in TCGA [24]. As shown in Fig. 3, this tool permitted us to analyze the methylation of ID4 tested with 13 probes distributed in different regions of the gene (the localization of each probe is represented in the figure and the ones localized in the promoter region are highlighted in dark blue). As can be observed, the methylation values increase as tumors become ER+; this can also be observed in Additional file 3: Figure S3 for CpGs in the promoter of ID4. All the regions analyzed presented a negative correlation with respect to ID4 gene expression (Pearson’s correlation coefficients for each probe are indicated on the right), suggesting that ID4 methylation silences gene expression.

Another interesting observation is that ER status and ID4 expression present an inverse correlation, where ID4 expression gradually diminishes (due to promoter methylation) as tumors become ER+ (*p* < 0.0001). The MEXPRESS tool also allowed us to visualize ID4 expression and methylation status according to PAM50 breast cancer molecular classification. As observed in Fig. 3 the methylation of ID4 increases (and ID4 expression decreases) in luminal A and luminal B subtypes (both ER+ and represented as green lines in Fig. 3) whereas methylation decreases (and expression increases) in basal-like and normal-like subtypes (both ER− and represented in Fig. 3 as yellow and blue lines respectively). Since aberrant DNA methylation of promoter regions is one of the mechanisms for the silencing of tumor suppressor genes in cancer, and because ID4 promoter is mostly methylated in ER+ tumors, we can again speculate that ID4 behaves principally as a tumor suppressor gene in these groups of breast tumors.

### ID4 expression is associated to the expression of different genes according to ER status

**Fig. 4:**
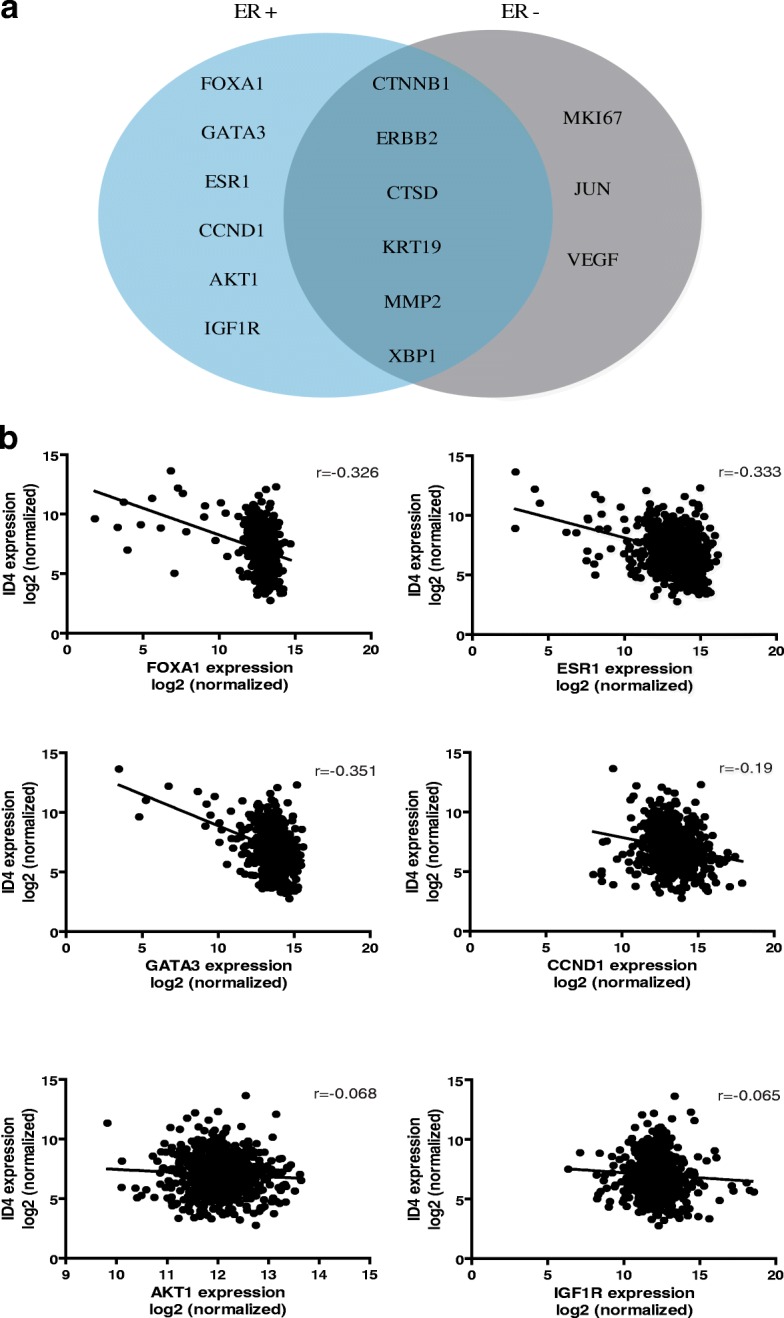
Genes associated with ID4 expression. **a** Venn diagram representing the expression for genes significantly associated with the expression of ID4 in breast cancer as determined by SVD analysis. The figure depicts the genes significantly associated with ID4 in ER+ and ER− tumors and the overlap the genes with shared expression between the two groups. **b** Correlations between ID4 expression and the expression of FOXA1, ESR1, GATA3, CCND1, AKT, and IGF1R. Correlation values for each analysis are indicated on the right

We next expanded our analysis to investigate whether the expression of ID4 is associated with different genes according to the ER status. To test this, we used the singular-value decomposition (SVD) analysis and studied the expression of ID4 vs. the expression of 66 genes with different functions in breast cancer (Table 1) in 780 samples from TCGA database. SVD represents an appropriate tool for gene expression analysis in which the singular values are associated with the importance of each variable in the linear system. The SVD program we used for this analysis is not a commercial one, such as MATHLAB, because these programs sort the singular values after they are calculated, and as a consequence, one loses the correspondence between ID4 and the gene under study. Rather, we used a C++ program written by one of the authors that leaves the singular values unsorted and hence maintains this correspondence. To define the most relevant genes associated with ID4 expression, we selected the genes that were higher than the median for the 66 singular values; 15 of the 66 genes met this criterion, which differed depending on the ER status. In ER+ tumors, ID4 expression was associated with the expressions of FOXA1, GATA3, ESR1, CCND1, AKT1, and IGFR; whereas in ER− tumors, ID4 expression was associated with VEGF, JUN, and MKI67. Finally, shared association was found for CTNNB1, ERBB2, CTSD, KRT19, MMP2, and XBP1 (Fig. 4a).

**Table 1:** Genes involved with distinct functions in breast cancer

Gene symbol	Gene name	Molecular and cellular function
ABCB1 (MDR1)	ATP-binding cassette subfamily B member 1	Xenobiotic transport
ABCG2 (BCRP)	ATP-binding cassette subfamily G member 2	Xenobiotic transport
ADAM23	ADAM metallopeptidase domain 23	Proteolysis
AKT1	AKT serine/threonine kinase 1	Signal transduction (AKT and PI3 kinase signaling)
APC	Adenomatosis polyposis coli	Signal transduction (WNT signaling), cell adhesion, apoptosis, cell cycle, DNA damage, and repair
AR	Androgen receptor	Signal transduction (steroid receptor-mediated signaling) and transcription factor
ATM	ATM serine/threonine kinase	DNA damage and repair
BCL2	BCL2, apoptosis regulator	Signal transduction (hedgehog signaling), cell adhesion, apoptosis, and cell cycle
BIRC5	Baculoviral IAP repeat-containing	Signal transduction (Notch signaling)
BRCA1	Breast cancer 1	DNA damage and repair and signal transduction (steroid receptor-mediated signaling)
BRCA2	Breast cancer 2	DNA damage and repair
CCNA1	Cyclin A1	Cell cycle
CCND1	Cyclin D1	Cell cycle, DNA damage and repair, and signal transduction (hedgehog and WNT signaling)
CCND2	Cyclin D2	Cell cycle
CCNE1	Cyclin E1	Cell cycle and signal transduction (steroid receptor-mediated signaling)
CDH13	Cadherin 13	Cell adhesion and angiogenesis
CDK2	Cyclin-dependent kinase 2	Cell cycle
CDKN1A	Cyclin-dependent kinase inhibitor 1A	Cell cycle, DNA damage and repair, and apoptosis
CDKN1C	Cyclin-dependent kinase inhibitor 1AC	Cell cycle
CDKN2A	Cyclin-dependent kinase inhibitor 2A	Cell cycle, apoptosis, and cell adhesion
CST6	Cystatin E/M	Proteases
CTNNB1	Catenin beta 1	Signal transduction (steroid receptor-mediated signaling), epithelial to mesenchymal transition, angiogenesis, and cell adhesion
CTSD	Cathepsin D	Proteases
EGF	Epidermal growth factor	Angiogenesis
ERBB2	Erb-b2 receptor tyrosine kinase 2	Signal transduction (AKT/PI3K signaling), angiogenesis, and cell adhesion
ESR1	Estrogen receptor 1	Signal transduction (steroid receptor-mediated signaling) and transcription factor
ESR2	Estrogen receptor 2	Signal transduction (steroid receptor-mediated signaling) and transcription factor
FOXA1	Forkhead box A1	Transcription factor
GATA3	GATA-binding protein 3	Transcription factor
HIC1	HIC ZBTB transcriptional repressor 1	Transcription factor
ID1	Inhibitor of DNA binding 1	Angiogenesis and breast cancer metastasis to lung and breast cancer classification marker
IGF1	Insulin-like growth factor 1	Signal transduction (steroid receptor-mediated and AKT/PI3K signaling)
IGF1R	Insulin-like growth factor 1 receptor	Signal transduction (AKT/PI3K signaling)
IGFBP3	Insulin-like growth factor-binding protein 3	Signal transduction (glucocorticoid signaling)
IL6	Interleukin 6	Angiogenesis and apoptosis
JUN	Jun proto-oncogene	Angiogenesis, apoptosis, cell cycle, and transcription factor
KRT19	Keratin 19	Signal transduction (steroid receptor-mediated signaling)
MAPK1	Mitogen-activated protein kinase 1	Signal transduction (MAP kinase-mediated signaling) and DNA damage and repair
MAPK3	Mitogen-activated protein kinase 3	Signal transduction (MAP kinase-mediated signaling)
MAPK8	Mitogen-activated protein kinase 8	Signal transduction (MAP kinase-mediated signaling)
MGMT	O-6-methylguanine-DNA methyltransferase	DNA damage and repair
MKI67	Marker of proliferation Ki-67	Cell cycle
MLH1	MutL homolog 1	DNA damage and repair
MMP2	Matrix metallopeptidase 2	Proteases and breast cancer metastasis to lung and breast cancer classification marker
MMP9	Matrix metallopeptidase 9	Proteases
MYC	V-myc avian myelocytomatosis viral oncogene homolog	Cell cycle and transcription factor
NME1	NME/NM23 nucleoside diphosphate kinase 1	Signal transduction (glucocorticoid signaling) and apoptosis
NOTCH1	Notch 1	Signal transduction (Notch signaling) and angiogenesis
NR3C1	Nuclear receptor subfamily 3 group C member 1	Signal transduction (glucocorticoid signaling) and transcription factor
PGR	Progesterone receptor	Signal transduction (steroid receptor-mediated signaling) and transcription factor
PLAU	Plasminogen activator, urokinase	Angiogenesis and proteases
PRDM2	PR/SET domain 2	Transcription factor
PTEN	Phosphatase and tensin homolog	Signal transduction (AKT/PI3K signaling), angiogenesis, cell adhesion, and cell cycle
PYCARD	PYD and CARD domain-containing	Proteases
RARB	Retinoic acid receptor beta	Apoptosis and transcription factor
RASSF1	Ras-association domain family member 1	Cell cycle
RB1	RB transcriptional corepressor 1	Signal transduction (steroid receptor-mediated signaling), cell cycle, and transcription factor
SERPINE1	Serpin family E member 1	Angiogenesis
SFN	Stratifin	Apoptosis, cell cycle, and DNA damage and repair
SFRP1	Secreted frizzled-related protein	Signal transduction (WNT signaling) and apoptosis
SLIT2	Slit guidance ligand 2	Angiogenesis
THBS1	Thrombospondin 1	Angiogenesis and cell adhesion
TP53	Tumor protein p53	Apoptosis, cell cycle, DNA damage and repair, and transcription factor
TP73	Tumor protein p73	Apoptosis, DNA damage and repair, transcription factor, and signal transduction (MAP kinase signaling)
VEGFA	Vascular endothelial growth factor	Angiogenesis
XBP1	X-box binding protein 1	Transcription factor

Next, we focused on ER+ tumors to determine the type of correlation between ID4 and the specific genes of interest within this group. By calculation of the correlation coefficients, we found that ID4 expression is negatively correlated with the expression of FOXA1 (*r* = − 0.326) (*p* < 0.0001), GATA3 (*r* = − 0.3515) (*p* < 0.0001), ESR1 (*r* = − 0.333) (*p* < 0.0001), CCND1(*r* = − 0.19) (*p* < 0.0001), AKT1 (− 0.068) (*p* = 0.094), and IGFR (− 0.065) (*p* = 0.1) (As shown in Fig. 4b). These negative correlations maintain their significance even when all the tumor cohorts (ER+ and ER−) are analyzed (Additional file 4: Figure S4). These results are particularly interesting given that some authors suggest that ID4 inhibits the expression of ESR1 and FOXA1 (through the interaction of ID4 with the promoter of these genes) in the developing mammary gland [29]. We hypothesize then that in an ER+ context, ID4 downregulation, trough methylation, disrupts the normal balance of important genes of the ER pathway.

Taken all together, from our in silico analyses, we can so far propose that in ER+ breast tumors, ID4 behaves as a tumor suppressor gene epigenetically regulated by DNA methylation.

### Ectopic ID4 expression reduces aggressive phenotype only in ER+ breast cancer cell lines

**Fig. 5:**
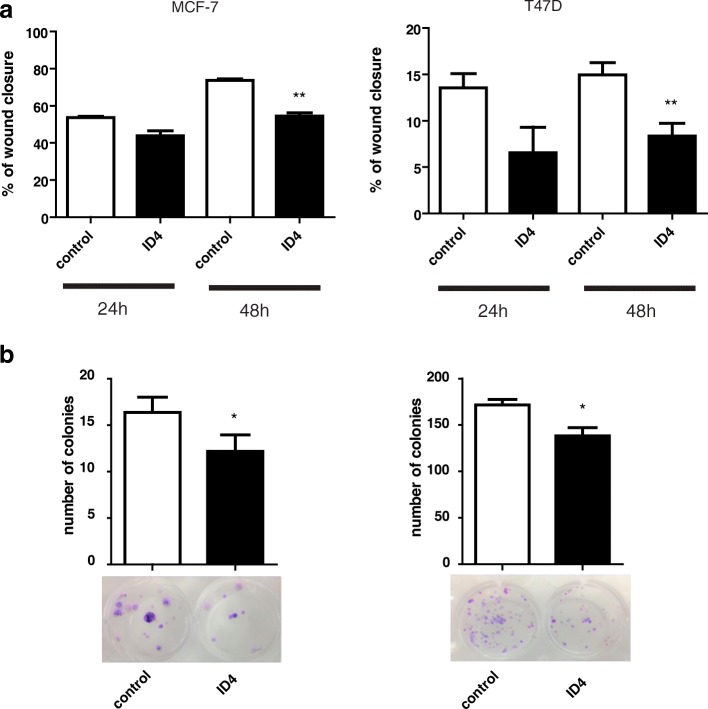
Phenotypic changes associated with ectopic ID4 expression in breast cancer cell lines. **a** Bar graph presentation of wound healing assay comparisons. The effect of ectopic expression of ID4 was tested on cell migration ability in MCF-7 and T47D cell lines (left and right respectively). Columns represent the mean of at least three independent experiments. *p < 0.05 and **p < 0.01 in comparison with the control per Student’s *t* test. For migration experiments, cells were maintained in a serum-reduced medium to inhibit the cells’ ability to proliferate. **b** Colony formation assay was used to confirm the effect of ID4 expression in T47D and MCF-7 transfected either with ID4 or the control vector. Results are expressed as the mean ± SD of three independent experiments

Based on our in silico conclusions, we decided to extend our studies and measure tumoral behavior by modulating the expression of ID4 in cultured ER+ breast cancer cell lines. To accomplish this, we performed transfection experiments with an ID4 vector in MCF-7 and T47D cells. We have previously shown that both cell lines do not express ID4 due to promoter methylation. [22]. Next, we measured cell migration potential (by wound healing assays) and colony formation ability, two hallmarks of cancer cells. As shown in Fig. 5a, both cell lines transfected with ID4 presented a significant reduction in migration rate compared to cells transfected with the control vector (*p* < 0.05). Consistently, colony formation assay showed that ID4 overexpression in MCF-7 and T47D cells led to a significant decrease in the number of colonies when compared with cells transfected with the control vector *(p* < 0.05) (Fig. 5b).

**Fig. 6:**
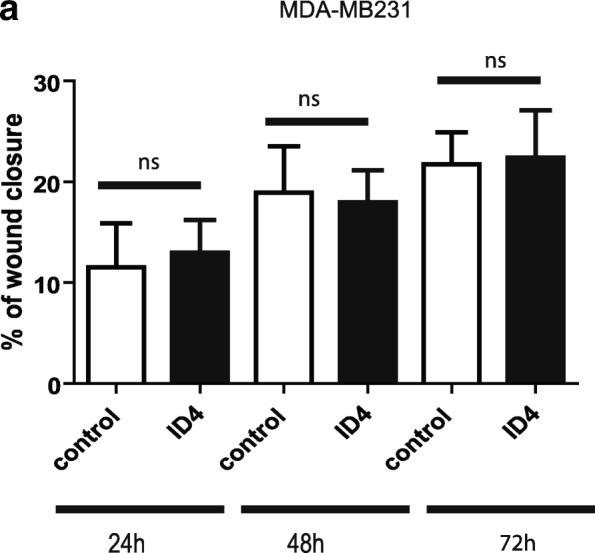
ID4 overexpression does not affect migration capacity in the ER− cell line MDA-MB231. The effect of ectopic expression of ID4 was tested on cell migration ability in the MDA-MB231 cell line. Columns represent the mean of at least three independent experiments

To confirm that ID4 behaves as a tumor suppressor only in ER+ breast tumors, we tested the effect of ID4 overexpression in the ER− breast cancer cell line MDA-MB231. To accurately study the effect of ID4 in an ER− context, we first confirmed by ddPCR that the MDA-MB231 cell lines did not express ER. Next, we performed transfection experiments with the ID4 vector as previously described and tested cell migration potential (by wound healing assays). ID4 overexpression did not affect migration capacity of the MDA-MB231 cell lines (Fig. 6).

Taken together, the transfection assays suggest concordantly that the ectopic expression of ID4 is inducing a less aggressive phenotype only in ER+ cell lines, revealed by a decreased migration and a reduced ability to produce new colonies. These results are in line with a dual role of ID4 in breast cancer.

### ID4 expression reduces ER levels in MCF-7 cells as assessed by ddPCR

**Fig. 7:**
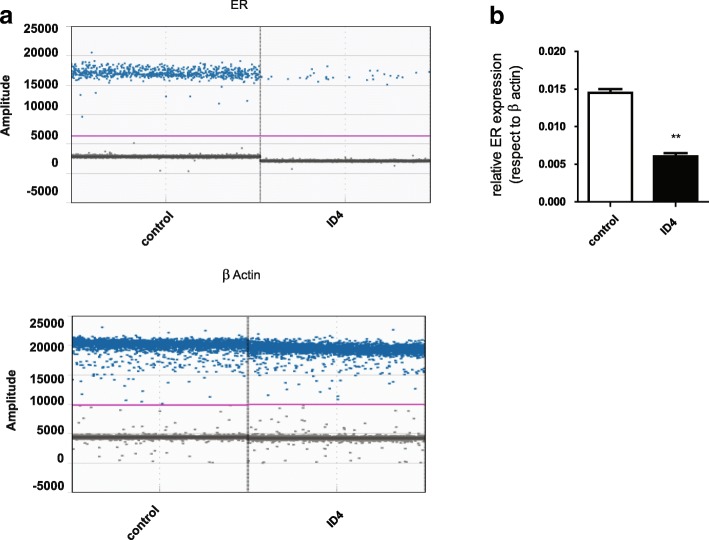
ID4 re-expression reduces ERα levels in MCF-7 cell lines. The figure represents the absolute quantification of ER expression (top left) and normalized respect to β-actin (right) by ddPCR. The fluorescence amplitude (y-axis) represents the intensity of amplification in each droplet, and each blue dot is a droplet in which the target has been amplified. To calculate the copies/droplet, a Poisson correction is performed which requires full (dost above the pink line) and empty droplets (dots below the horizontal pink line). For this, a minimum and equal amount of template cDNA (10 ng) is used for each condition. **a** With the ddPCR assay for ER expression, a variation was observed in the number of droplets with signal of ERα detection. β-actin was used as a control. Results are presented as copies per microliter in the amplification reaction. **b** Bar graph represents the mean of three technical experiments measuring the expression of ER in ID4-transformed MCF7 cells, by ddPCR. **p < 0.05 in comparison with the control per Student’s *t* test

As we mentioned previously, ID4 expression is associated with key genes of the ER pathway. Taking this observation into consideration, we decided to study the effect of ID4 overexpression on ER levels in an ER+ cell line. To test this, we transfected MCF-7 cells with the ID4-expressing plasmid. As control, cells were transfected with the GFP control vector. When obtaining total RNA from both experiments and measuring the expression level of ER by ddPCR, a significant difference could be observed. As shown in Fig. 7, ID4 overexpression induced a significant reduction in ER levels of MCF-7 cells as compared to the same cells transfected with a control vector (*p* < 0.01).

Given that in ER+ breast, tumors ER levels are higher than in normal tissue, and these higher levels are associated with certain aggressive characteristics such as increased cell proliferation, and we speculate that ID4 re-expression in ER+ cell lines reduces the ER levels perhaps to those of normal tissue and possibly exerts its tumor suppressor function trough ER regulation (Fig. 7).

### ER levels do not affect methylation nor ID4 expression levels in MCF-7 cell lines

**Fig. 8:**
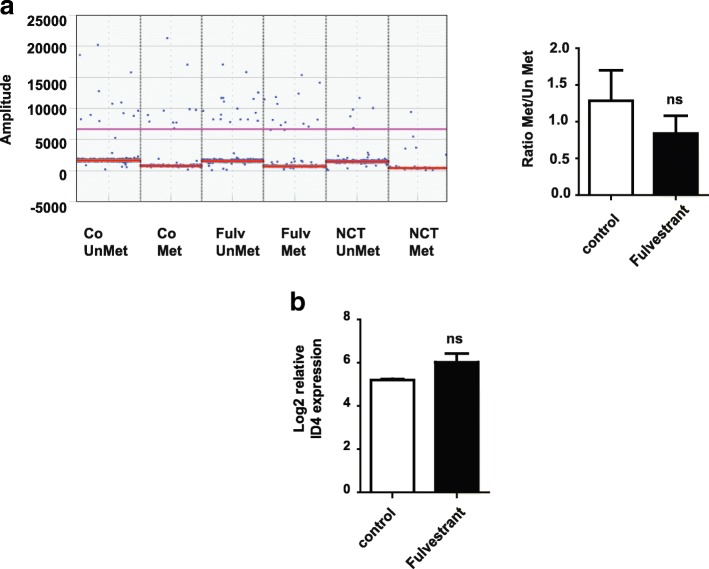
Estrogen deprivation does not affect neither ID4 methylation nor ID4 expression. **a** Left, ddPCR assay for the detection of ID4-methylated status in control and fulvestrant-treated MCF-7 cells. Results are presented as copies per microliter in the amplification reaction. Right, bar graph represents the mean of three technical experiments measuring the methylated/unmethylated ratios of the control and experimental conditions. **b** ID4 probe (A_23_P59375) intensity was compared between control and fulvestrant-treated MCF-7 cells

To study if there is a regulatory loop between ER and ID4, we next tested if estrogen deprivation affected the methylation status or the expression levels of ID4 in the ER+ cell line MCF-7. To test this, we first treated the cells with 1μM of the ER antagonist fulvestrant and measured ID4 methylation status by ddMSP and MS-MLPA. As shown in Fig. 8a, there was a slight reduction in the methylation levels of fulvestrant treated cells, but this difference was not statically significant (*p* = 0.45). To further analyze if there were changes in the methylation status of other regions of ID4 promoter, we performed a MS-MLPA assay with the ME003 panel. This panel contains 27 probes two of which hybridize at different CpG sites from that tested by ddMSP. The MS-MLPA assay revealed that there was no significant difference in the methylation level between control and fulvestrant-treated cells in neither of the CpG sites tested (Additional file 5: Figure S5).

Since it has been shown that promoter methylation is the main mechanism that controls ID4 gene expression [13, 17, 28], we speculated (given our ddMSP and MS-MLPA results) that there was not going to be differences in ID4 gene expression after estrogen deprivation. To confirm this, we performed in silico analysis on a public data set of an in vitro model of MCF-7 cells treated with ER antagonists such as fulvestrant (ArrayExpress accession number E-MTAB-4426), and as shown in Fig. 8b, there were no differences on ID4 gene expression between control and fulvestrant-treated cells.

Taken together, our results reveal that while ID4 reduces ER expression, estrogen levels do not affect neither ID4 methylation nor ID4 expression. These observations suggest that there seems not to be a regulation of estrogen towards ID4.

## Discussion

Given a certain cellular context, ID proteins may follow divergent functions and act as tumor suppressors or as oncogenes [2]. Particularly, ID4 can act as a tumor suppressor and as an oncogene in different tumor types, e.g., prostate, gastric, glioblastomas, and colorectal tumors [1, 11, 13, 28–30]. In breast cancer, our group and others have previously shown that ID4 may behave as an oncogene in TNBC or basal-like breast tumors. Principally in ER− tumors, the role of ID4 has been linked to BRCA1 downregulation and BRCAness phenotype [21, 22, 31]. But other authors have shown that ID4 may also act as a tumors suppressor in breast cancer [17, 32], where there seem to be controversial findings regarding the role of ID4. We hypothesize that ID4 behaves as both a tumor suppressor and an oncogene as well and that the difference in behavior varies according to the ER status of breast tumors.

In this report, we used two approaches to test our hypothesis: data mining analysis and in vitro experiments. Data mining analysis revealed that ID4 expression is significantly downregulated in ER+ breast tumors as compared with ER− tumors or normal tissue. We show here that ID4 is silenced in breast tumors through promoter methylation and that ID4 is methylated as tumors become ER+. Interestingly, ID4 expression is significantly higher in normal tissue with respect to breast tumors either they are ER+ or ER−. This could be indicating that ID4 expression is required for normal mammary function and that during a tumorigenic process (either ER+ or ER−), ID4’s expression is reduced through methylation. Comparing ER+ with ER− tumors, the methylation levels are not the same. Therefore, there are different expression levels of ID4 between these tumor types and possibly different pathways are turned on or off according to differences in ID4 expression. Some authors suggest that ID4 regulates linage commitment and forms part of a complex regulatory network with ERα and BRCA1 in the normal mammary gland [18]. Perhaps during mammary tumorigenesis, ID4 follows divergent pathways in ER+ and ER− tumors, affecting different cellular networks. Previously published work shows that specific binding of ID4 (as part of a larger complex) occurs at a region located 5.9 kb upstream of the ERα promoter [29]. Here, we show that ID4 re-expression in MCF-7 cells induced a significant reduction in ER α expression. We also demonstrate by in silico analysis that ID4 expression is associated with different genes according to ER status. In ER+ tumors, ID4 expression was negatively correlated to the expression of key genes of the ER pathway such as FOXA1, GATA3, ESR1, and CCND1. Interestingly, all the four genes are involved in the ER pathway. An ID4 site has been identified 8.3 kb upstream of the FOXA1 transcription start site and perhaps forms part of a similar protein complex [29]. Taking these observations into consideration, we could speculate that in the normal mammary gland, ID4 is an important member of the ER pathway and when its expression is affected by methylation, and in ER+ tumors, the expression of important players of the ER pathway is disturbed.

The observation that the expression of ID4 associates with VEGF, JUN, and MKI67 in ER− tumors has not been previously described in breast cancer and it reveals that the involved pathways differ depending on the ER status.

We also evaluated the effect of ID4 expression on OS in breast cancer patients. We observed that in ER+ breast tumors, high ID4 expression was associated with better probabilities of survival. This was not observed among ER− tumors, where the expression of ID4 does not correlate with OS. It is worth mentioning, however, that in the basal-like subgroup of ER− tumors, the expression of ID4 did show a positive association with worst survival (*p* = 0,043). Even though we do not yet understand why this subgroup presents this behavior, it supports the hypothesis of a dual role for ID4 based on the ER status.

Finally, the putative tumor suppressor function of ID4 in ER+ breast cancer was verified by in vitro assays. ID4 overexpression induced phenotypic changes associated with a tumor suppressor role for this protein in ER+ breast cancer cell lines. This was evidenced by reduced migration rates in both breast cancer cell lines analyzed. The reduction in migration could be related to the fact that we found ID4 associated with CCND1, which regulates migration and proliferation in breast cancer cells [33–35]; perhaps, these changes in migration are due to an interaction between ID4 and CCND1. This would not be the first case reporting an association between a member of the ID family with CCND1. For instance, Tobin et al. established that there exists a relationship between cyclin D1, ID1, and EMT in primary breast cancer [36]. Ours is the first report suggesting an association between ID4 and CCND1 in breast cancer. Further research should be conducted to confirm this data. Our observations that ectopic expression of ID4 also leads to a significant decrease in the number of colonies is also in line with a tumor suppressor role for this protein.

## Conclusions

We propose that ID4 is frequently silenced by promoter methylation in ER+ breast cancers and functions as a tumor suppressor gene in these tumors, probably because of its negative interaction with key genes of the ER pathway. Our present study contributes to the knowledge of the role of ID4 in breast cancer.

## Additional files

Additional file 1: **Figure S1.** qPCR and Western blot analysis of ID4 expression in control and transfected cells with ID4. (AI 1152 kb)

Additional file 2: **Figure S2.** Higher ID4 expression is associated with OS in ER+ tumors. Kaplan–Meier analysis of the Miller 2005 cohort evaluating OS in ER+ tumors. (AI 1076 kb)

Additional file 3: **Figure S3.** ID4 methylation values according to ER status. Box plot representation of *β* values of ID4 methylation at different sites in ID4 promoter. The bottom and top of the box represent the first and third quartiles of the data, respectively, and the band inside the box represents the median of the data. The lower and upper whiskers represent the lowest and highest data points of the data, respectively. ***p < 0.001. (AI 1193 kb)

Additional file 4: **Figure S4.** Genes associated with ID4 expression in ER+ and ER− tumors. Correlations between ID4 expression and the expression of FOXA1, ESR1, GATA3, CCND1, AKT, and IGF1R in the complete tumor cohort (ER+ and ER−). Correlation values for each analysis are indicates on the right. (AI 1561 kb)

Additional file 5: **Figure S5.** MS-MLPA analysis of ID4 status after fulvestrant treatment. MS-MLPA ME003 probemix was used to analyze ID4 status after estrogen deprivation in MCF7 cells. (AI 1416 kb)

## Abbreviations

bHLH: Basic helix-loop-helix
ER: Estrogen receptor
ID: Inhibitor of differentiation
KM: Kaplan–Meier
OS: Overall survival
SVD: Singular-value decomposition
TCGA: The Cancer Genome Atlas
